# Going Gentle Into That Good Night

**Published:** 2015-01-01

**Authors:** Pamela Hallquist Viale

As a result of continued improvements in the treatment and detection of cancer, it is estimated that the population of cancer survivors will increase to approximately 19 million by the year 2024 (American Cancer Society, 2014). Healthier lifestyles undoubtedly contribute to that number as well. This is a remarkable achievement in our battle against this formidable disease. But what do we do when we can’t win the battle and the troops may be ill equipped to manage the retreating patient who hasn’t any viable options left?

## THE CURRENT HEALTH-CARE APPROACH TO DYING

I have recommended Atul Gawande’s book *The Checklist Manifesto: How to Get Things Right* in the pages of *JADPRO* before; he is an uncommonly sane voice on what’s wrong in medicine and what we can do to improve our care. His latest book, *Being Mortal: Medicine and What Matters in the End*, confronts our medical care priorities and is a cogent exploration into mortality and what is most important at the end of life. The American health-care system spends a staggering amount of money on the care of patients who are essentially at the end of life, employing treatment measures that are often useless, invasive, and very expensive (Gawande, 2014).

## PERSONAL CHOICES

I find Dr. Gawande’s viewpoint refreshing and reassuring. You see, I had to face the challenges he addresses when I took care of my mother in her last year of life. Diagnosed with terminal lung cancer, she received radiation therapy to combat the metastatic lesions in her brain. The treatments left her almost childlike and dependent on me for her care. I took a year off of work to care for her at home and tried to fill her days with excursions and things that I knew she had loved during her lifetime. We went to plays, we visited Napa wine country, and we took a train trip. We tried to visit with special family members. Her ability to read had unfortunately diminished after her radiation therapy treatments, but she still loved turning the pages of cookbooks, eagerly looking at the pictures of the food and picking out dishes for me to cook for her.

In short, I tried to fill my mother’s days with the things she’d enjoyed most. Earlier on in her journey, she had stated that she wanted to end her life at home, with her family. When she started to slip into a semi-comatose state one morning, I did NOT take her to the emergency room for more radiation therapy and the massive doses of steroids that might have reversed the acute problem but would not have been able to reverse the incurable metastatic tumor in her brain. Three days later, she died at home in my arms with my brother at her side.

## ALTERING OUR APPROACH TO THE DYING PATIENT

In *Being Mortal*, Dr. Gawande shares personal stories of his own family members who faced death in different ways: some successfully, yet some not so successfully, dying in very uncomfortable circumstances. He talks about health-care professionals who have worked to alter our approach to end-of-life care and the dying patient, including our colleagues in hospice and palliative care, who help those patients achieve a dignified and comfortable death.

Dr. Gawande outlines four important questions that clinicians can use with their patients so that expectations are outlined and accepted and end-of-life care preferences are prioritized. These questions are simple and direct, but I think they could be so very helpful when caring for patients who are at the end of their lives: (1) What is your patient’s understanding of their health or condition? (2) If their health worsens, what are their goals? (3) What are they afraid of? (4) What tradeoffs are they are willing or not willing to make? (Gawande, 2014).

These are extremely important questions, yet they are not always integrated into our standard of care for those patients who have no further viable treatment options. I think it is easy to see why clinicians might avoid discussing end of life. My mother was told of her incurable disease, but with her somewhat limited cognition, we needed to frequently reinforce her knowledge and acceptance of that fact. It was often depressing to talk about it again and again. We knew her goals: She wanted to stay at home and try to do things she enjoyed as much as possible. Her fears were the universal ones: She wasn’t ready to die, but she understood the futility of further therapy. The tradeoffs were easy. She gave up further unnecessary, futile, and expensive therapies for quality of life at home. However, my mother was incredibly lucky. Her daughter and caretaker was an oncology nurse practitioner who could explain what extraordinary measures were and help her understand their often pointless results.

## FINAL THOUGHTS

I cannot recommend Dr. Gawande’s book enough. Oncology health-care professionals will surely relate to the difficulties encountered by patients facing their mortality. The four questions he puts forth should—and in fact must—be answered to contribute to an improved and more acceptable death. Perhaps the four questions will become part of the curriculum for advanced practitioners taking care of our patients with incurable disease who are facing mortality. Preserving life at all costs is not always the answer. Death will eventually come to everyone. For many of our patients, death will come sooner rather than later. As advanced practitioners in oncology, we must constantly work on our ability to help patients achieve that inevitable end gently and on their own terms.

## References

[1] (2014). Cancer treatment and survivorship facts & figures 2014-2015.. *Atlanta, Georgia: American Cancer Society.*.

[2] Gawande A. (2014). *Being mortal: Medicine and what matters in the end.*.

